# Should We Rely on Screening Tests for Further Management Alone in Polymerase Chain Reaction Negative COVID-19 Patients? A Case Series

**DOI:** 10.7759/cureus.10555

**Published:** 2020-09-20

**Authors:** Phool Iqbal, Fateen Ata, Samman Rose, Hammad S Chaudhry, Ali Rahil

**Affiliations:** 1 Internal Medicine, Hamad General Hospital, Doha, QAT; 2 Internal Medicine, Hamad Medical Corporation, Doha, QAT

**Keywords:** covid 19, sars-cov-2 (severe acute respiratory syndrome coronavirus -2), sars-cov-2, corona virus disease 2019, real time-pcr, serology

## Abstract

Since the declaration of coronavirus disease 2019 (COVID-19) disease as a pandemic by the World Health Organization (WHO), it has been a challenge to the whole medical community. Researchers and clinicians have been trying to explain and explore its mechanism and pathophysiology to get a better understanding of this disease, as it has exhausted the healthcare resources and has impacted human life in general. Many tests have been developed including polymerase chain reaction (PCR) of the virus and rapid diagnostic testing in patients based on IgM/IgG serology. But owing to variable sensitivity and specificity of these tests, it has created a challenging situation to proceed with the further management plan. We are reporting a case series where we experienced the dilemma of diagnosing COVID-9 disease in our patients and further plan of care.

## Introduction

The coronavirus disease 2019 (COVID-19) outbreak was declared a worldwide pandemic by the World Health Organization (WHO) on 11 March 2020 [1]. Because this disease is new to the medical community, there have been challenges in diagnosing and treating its various clinical manifestations. Currently, the ongoing research has resulted in many published research articles that can provide some insight, but the dilemma, related to the diagnostic testing for the virus and further management plans, prevails. The GeneXpert express system technology uses the polymerase chain reaction (PCR) to detect the severe acute respiratory syndrome-coronavirus-2 (SARS-CoV-2) obtained from nasopharyngeal swabs. Herein, we report our experience with COVID-19 patients who were tested multiple times using this system but were negative for the SARS-CoV-2 virus. However, improvement was observed in the clinical condition of the patients who were managed as per COVID-19 protocol based upon the clinical signs and symptoms after correlating with diagnostic chest imaging studies.

## Case presentation

Case 1

A 37-year-old Nepalese man, with hypertension that was controlled by medications, presented with a two-day history of fever and myalgia. There was no history of any recent travel or contact with an ill person.

On the initial presentation, his clinical examination was remarkable, with a high-grade temperature of 39°C. He was breathing comfortably in room air, and a respiratory system examination indicated that he was normal. Due to the ongoing COVID-19 pandemic, a nasopharyngeal swab sample was obtained, and he was tested for COVID-19 using PCR. The test was negative for the SARS-CoV-2 virus, and thus, he was discharged home with symptomatic treatment, and no further investigations were performed.

Ten days later, he presented with a five-day history of fever, cough, and generalized body aches. This time, the patient’s breathing rate increased to 24 breaths per minute, with tachycardia of 100-110 beats per minute, oxygen saturation of 94%-95% in room air, and bilateral basal crackles on chest auscultation.

**Figure 1:**
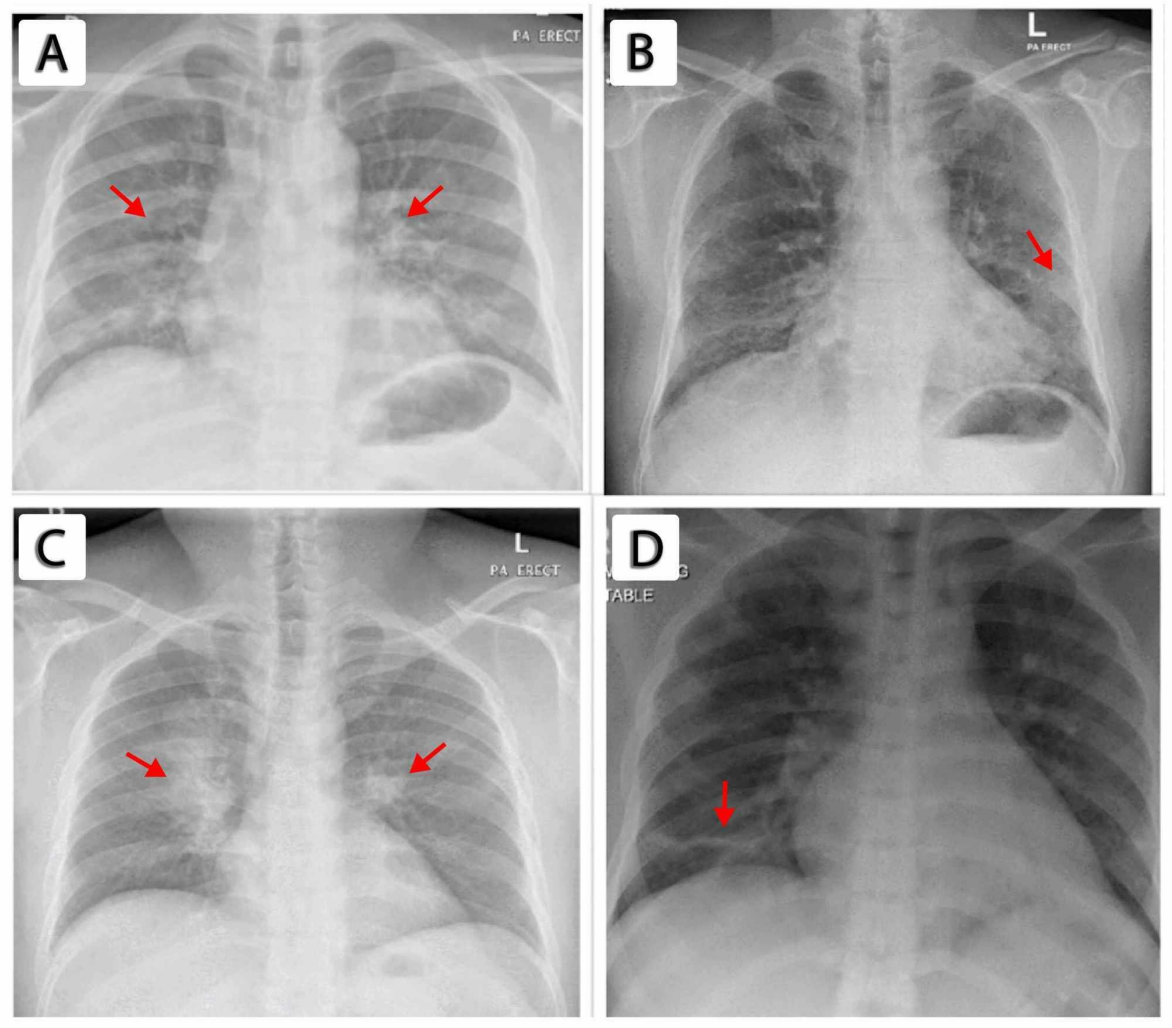
Chest X-rays of patients. (A) Patient 1 exhibiting bilateral patchy infiltrates and nonhomogenous opacities located mostly in the lower zones (red arrows). (B) Patient 2 exhibiting bilateral patchy infiltrates and nonhomogenous opacities located in the peripheral areas (red arrows). (C) Patient 3 exhibiting bilateral patchy infiltrates and nonhomogenous opacities more prominent in the middle zones (red arrows). (D) Patient 4 exhibiting bilateral patchy infiltrates and nonhomogenous opacities located peripherally (red arrows).

His chest X-ray (CXR; Figure 1A) showed bilateral middle and lower zone opacities, and treatment was initiated with ceftriaxone and azithromycin for community-acquired pneumonia. However, his condition did not improve. His COVID-19 PCR test was subsequently repeated an additional two times from nasopharyngeal swab samples, and the test was negative both times. Septic workup, including blood and sputum culture, tuberculosis (TB) workup including sputum smear, and urine culture were negative. Laboratory investigations were remarkable for lymphopenia, high D-dimer, C-reactive protein (CRP), and ferritin. The infectious disease team advised testing with COVID-19 serology (immunoglobulin (Ig) M and IgG antibodies through lateral flow assay), the results of which were positive, indicating recent infection. He was maintained on isolation protocols for COVID-19, and treatment was administered based on local guidelines. He improved during his hospital stay with no further clinical deterioration and was discharged home with follow-up in the outpatient department.

Case 2

A 57-year-old Bangladeshi man with type 2 diabetes presented to the hospital with a dry cough and fever lasting for four days. He initially sought treatment from a private hospital, where he received oral antibiotics. Due to the worsening of his symptoms, he presented to the ED of our hospital. On clinical examination, he maintained oxygen saturation in room air but was in respiratory distress with mild tachypnea of 20-22 breaths per minute. His respiration system examination was unremarkable, although his CXR (Figure 1B) revealed basal nonhomogeneous opacities. Laboratory investigations were significant for elevated inflammatory markers, specifically, CRP, ferritin, and D-dimer.

His blood cultures and PCR test for respiratory viruses (adenovirus, influenza viruses, par influenza viruses, Rous sarcoma virus (RSV), human rhinovirus, enterovirus, and other coronaviruses, including Middle East Respiratory Syndrome (MERS) coronavirus and SARS-CoV-2), were negative. During the hospital stay, multiple nasopharyngeal swab samples were obtained, and PCR tests were performed to detect COVID-19 on the swabs. Five of the repeated tests were negative, and one was inconclusive. The infectious disease team was consulted and based upon his clinical presentation and previous investigations, the patient was maintained on the local management protocol for COVID-19 infection. Clinical improvement was noted, and then, he was discharged home without any complications.

Case 3

A 34-year-old Nepalese man with no past medical history presented with fever, dry cough, and sore throat lasting for five days. Upon presenting to the ED, he maintained normal oxygen saturation at room air, but 24 hours later, he required 2 L/min of oxygen through a nasal cannula to maintain his oxygen saturation above 94%. His body temperature was higher than 38°C. His CXR (Figure 1C) showed ill-defined opacity in the right middle and lower zones in the perihilar region. The complete respiratory viral panel (adenovirus, influenza viruses, par influenza viruses, RSV, human rhinovirus, enterovirus, and other coronaviruses, including MERS coronavirus and SARS-CoV-2 nasopharyngeal swab) was negative. There was suspicion of Mycobacterium tuberculosis infection based upon CXR findings, but his TB workup (acid-fast bacillus smear and QuantiFERON test) was negative. Laboratory investigations were remarkable for high inflammatory markers, including CRP and ferritin. The PCR test for COVID-19 was performed four times from nasopharyngeal swabs. Three of the test results were inconclusive, while one was negative. Rapid serology (lateral flow assays) tests for IgM and IgG antibodies were positive and indicated recent infection.

The infectious disease team was consulted, and based on his clinical picture, he was managed based upon COVID-19 local treatment guidelines. He improved and did not require further oxygen supplementation. He was discharged home.

Case 4

A 34-year-old healthy Indian man without any past medical history came to the hospital with a high-grade fever of more than 38°C and a dry cough lasting for seven days. The patient was maintaining saturation on room air. CXR showed bilaterally patchy consolidation at the bases (Figure 1D). Septic workup was performed, consisting of PCR for respiratory viruses (including COVID-19) from a nasopharyngeal swab, blood cultures, TB workup, and urine culture. However, the results were unremarkable for any source of infection. His CRP and ferritin levels were high.

After two days, the patient required oxygen supplementation through a nasal cannula at 2-5 L/min. The infectious disease team was consulted, and the COVID-19 PCR test was repeated four times. The first two samples were negative, the third sample was inconclusive, and the fourth sample turned out to be positive. He was managed using the COVID-19 pneumonia local guidelines. His condition improved, and he did not require further oxygen.

## Discussion

Due to its highly contagious nature, SARS-CoV-2 has spread worldwide, and the COVID-19 global pandemic has adversely affected the economy, healthcare resources, and social interactions. The disease primarily affects the pulmonary system and may cause fever, cough, anosmia, loss of taste, and shortness of breath that may develop sufficient severity so that endotracheal intubation is required. Other various atypical presentations include diarrhea and hyponatremia [1-2]. Healthcare professionals across the world have been tailoring their diagnostic approach and treatment according to the local requirements and available limited evidence. The dilemma of a diagnosis based on less sensitive and specific tests or atypical presentations may cause complications due to delay in the management of COVID-19 patients.

Available tests

Reverse Transcription-Polymerase Chain Reaction

The reverse transcription-PCR (RT-PCR) test for COVID-19 is the most widely used method for screening purposes [3]. Although the sensitivity of this test is considered to be up to 90%, large-scale testing can lead to an increase in the number of false-negative cases, which contributes to further spreading of the virus in the community [3-4]. In one study, the sensitivity of the RT-PCR test for 205 patients was found to be highly variable, with 93% for bronchoalveolar lavage, 72% for sputum, 63% for nasal swabs, and only 32% for throat swabs. Moreover, the accuracy also depends upon certain other factors such as the stage of the disease, degree of viral multiplication or clearance, and if multiple genes are being tested [5]. Other tests currently used for suggestive COVID-19 infection are the rapid antibody detection test, CT of the chest, and biomarkers in the blood [4, 6-7]. Serology with rapid antibody detection has recently been introduced to estimate the immunity against COVID-19 infection, but the time course and accuracy of serology tests are still under investigation [7]. CT of the chest has been widely used due to typical changes related to COVID-19 infection that occur, (i.e., peripheral ground-glass opacities seen in the lungs) [8]. However, false-negative CT scan rates vary in the literature, ranging from 3% to 56% in RT-PCR-positive patients [6].

Artesi et al. highlighted this point in their study, where they assessed multiple RT-PCR methods and concluded that methods targeting a single genome could be unreliable [9]. One of their studied mutations was in the E gene. While using the Cobas® system (F. Hoffman-La Roche AG, Basel, Switzerland) for studying the reverse transcription of eight samples, it was found that they were negative for E gene reverse transcription but positive for ORF1ab reverse transcription. Four samples were retested by targeting the RdRP and E genes via the TaqMan Fast Virus 1-Step Master Mix (Thermo Fisher Scientific, Waltham, MA) SARS-CoV-2, which was described by Corman et al. [10].

Other Tests

Other tests currently used for suggestive COVID-19 infection are the rapid antibody detection test, chest CT examination, and biomarkers in the blood [4, 6-7]. Serology with rapid antibody detection has recently been introduced to estimate the immunity against COVID-19 infection, but the time course and accuracy of serology tests are still under investigation [7]. Chest CT has been widely used as well, due to typical changes related to COVID-19 infection; however, false-negative CT scan rates vary in the literature, ranging from 3% to 56% in RT-PCR-positive patients [6]. Therefore, the lack of a clear-cut gold standard test for COVID-19 infection creates a challenge for the medical community [5].

The idea of a pretest probability can be applied here. Pretest probability is high when a patient exhibits typical symptoms such as cough, fever, shortness of breath, history of exposure, and when a patient originates from a geographical location with a high prevalence of the disease [5]. Moreover, biomarkers such as CRP, ferritin, lymphocyte counts, lactate dehydrogenase, and N-terminal pro b-type natriuretic peptide, along with radiological findings in CXR or features such as unilateral or bilateral pneumonia, ground-glass opacities, or consolidations in a chest CT scan, can suggest COVID-19 infection even in such patients where RT-PCR alone is negative [4].

Treatment modalities

There have been inconsistencies in the guidelines for treating patients with COVID-19. Hydroxychloroquine, a drug used to treat malaria and systemic diseases such as rheumatoid arthritis and systemic lupus erythematosus, was effective in inhibiting the growth of the virus in in-vitro studies. Early data supported its role in the management of COVID-19 pneumonia [11]. However, it was associated with an increase in inpatient hospital mortality [12]. Anti-retroviral drugs, specifically, lopinavir and ritonavir, were also used for the treatment of COVID-19 infection [13]. Later, Cattaneo et al. found them to be ineffective in a prospective randomized, open-label trial [13]. As the pandemic progressed, in vitro experimentation led to the use of several drugs or their combination, including chloroquine, hydroxychloroquine, azithromycin, ribavirin, favipiravir, and interferons. However, promising results in improving survival have currently been obtained only with remdesivir and steroids, particularly dexamethasone [14]. In critical cases of COVID-19, where the patients exhibit hypoxia and high inflammatory markers, tocilizumab and convalescent plasma have also been used in combination with other drugs. However, as of 20 June 2020, there is no licensed medication for COVID-19 treatment [14].

Our experience with the patients

There have been numerous cases in our center, where the RT-PCR test for SARS-CoV-2 was performed multiple times, and the results were inconclusive repeatedly. Nevertheless, if the overall clinical picture concerning signs and symptoms is consistent with a probable viral respiratory infection, and laboratory tests indicate inflammation, then this is suggestive of SARS-CoV-2 infection. In these patients, acute symptoms of upper respiratory tract infection, such as sore throat, fever, shortness of breath, and myalgia, were suggestive of SARS-CoV-2 infection. High CRP, high ferritin, and D-dimer were measured in all patients, while lymphopenia was observed in one of the patients (Table 1). Although these are nonspecific findings, in the current literature, these findings are very likely to suggest SARS-CoV-2 infection [15].

**Table 1:** Laboratory investigation data. CRP, C-reactive protein; Ig, immunoglobulin.

Laboratory parameters	Reference range	Case 1	Case 2	Case 3	Case 4
CRP	0-5 mg/L	114 mg/L	227 mg/L	47.4 mg/L	89 mg/L
Ferritin	30-533 mg/L	871 mg/L	1335 mg/L	628 mg/L	622 mg/L
D-Dimer	0-0.44 mg/L	0.68 mg/L	0.59 mg/L	0.44 mg/L	Not performed
Serology (IgG/IgM)	+/-	+ (IgM and IgG)	Not performed	+ (IgM and IgG)	Not performed

We also performed septic and TB workups to rule out secondary infections that can present in the same manner. The results were unremarkable. However, CXR of all patients revealed bilateral nonhomologous opacities involving different zones of the lung field, which has been recently described in association with COVID-19 [6], as shown in Figure 1.

Based on the patients’ signs and symptoms, they were managed with isolation protocols and local guidelines. They improved within the COVID-19 recovery period based on mild to moderate pneumonia with no further deterioration during their hospital stay, and this further consolidated our diagnosis [16].

Critical analysis of the study

Positive COVID-19 RT-PCR indicates confirm COVID-19 case, but in our case 2, the patient did not test positive for RT-PCR and therefore labeled and managed as "probable COVID-19" due to the clinical picture and suggestive radiological imaging correlating with the infection [4]. On the other hand, the patients did not undergo CT scan imaging which is more sensitive than CXR. The rationale behind this is, that the CXR of all the patients was remarkable of unilateral/bilateral nonhomogenous opacities which is also attributed to COVID-19 [4]. And the laboratory findings were also suggestive. Serological testing is also helpful to suggest COVID-19 infection, however, it can be false-negative in an asymptomatic patient or who has been recently exposed but still, the body immune response did not develop sufficient detectable antibodies. In our cases, the patients were symptomatic, and positive serology testing indicates exposure and symptoms onset of more than five days [7].

Such cases can be easily missed in this pandemic state if a proper clinical and correlating investigational approach is lacking leading to unnecessary exposure to frontline health workers.

Proposed algorithm

**Figure 2:**
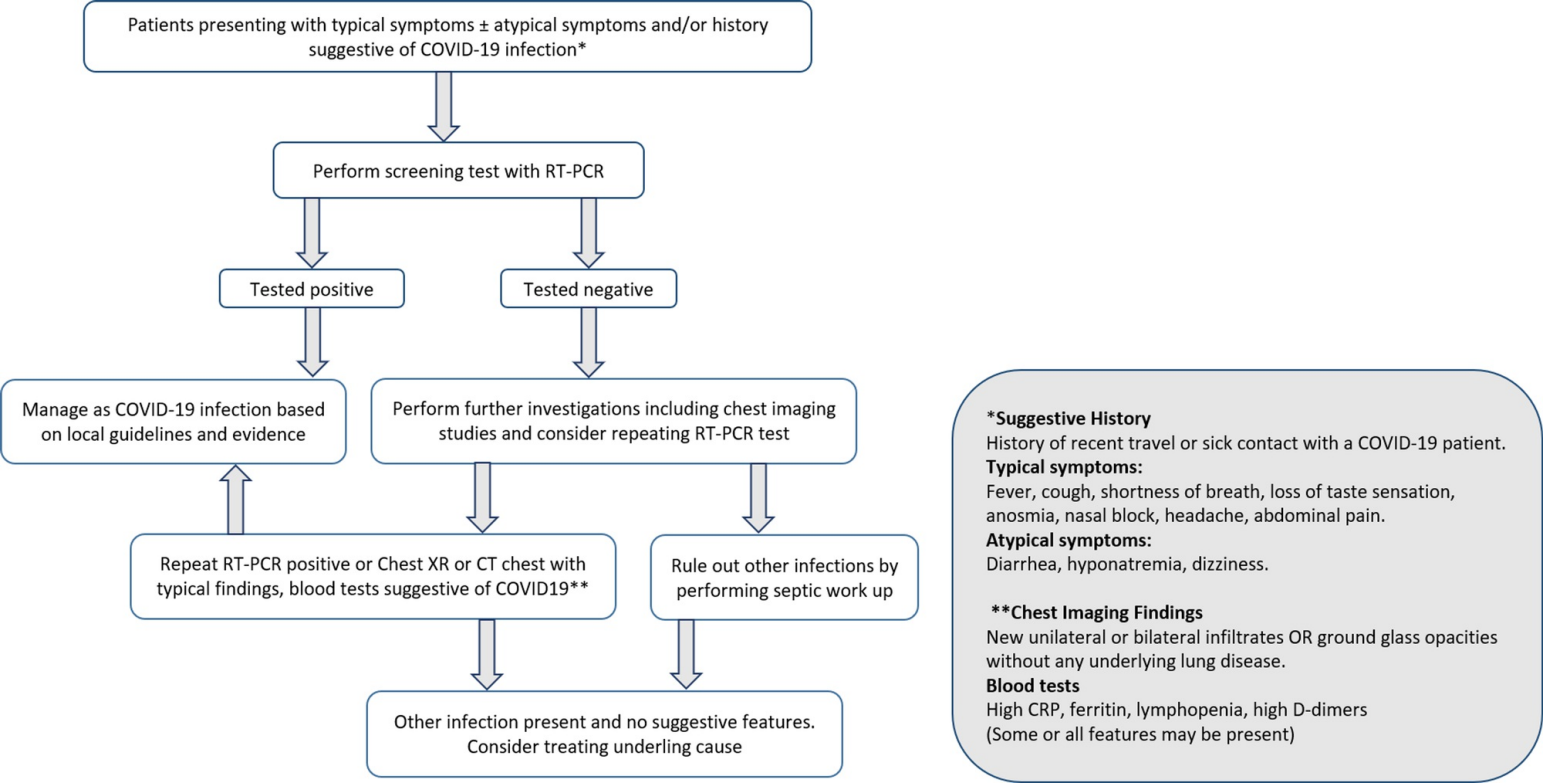
Proposed algorithm for highly suspected cases of COVID-19 that are negative for SARS-CoV-2 after performing the RT-PCR test. RT-PCR, reverse transcription-polymerase chain reaction; CRP, C-reactive protein.

Currently, a single test that can accurately detect SARS-CoV-2 infection does not exist. There are high false-negative rates even on repeated tests. There is a risk that SARS-CoV-2 will be spread in the hospital and community settings when false-negative test results are obtained. In such a situation, we should apply the idea of pretest probability based on the patient’s history, clinical signs and symptoms, suggestive biomarkers in the blood, and radiological imaging. Hence, after a literature review and taking into account our experience, we have proposed an algorithm to identify patients with high clinical suspicion of COVID-19 and to treat the patient for COVID-19 despite negative RT-PCR results (Figure 2) [6]. However, there is a need for larger-scale clinical studies to validate this pathway and establish it as a recommendation.

## Conclusions

The COVID-19 pandemic is a challenging global situation with various amounts of false-negative testing that occurs during the process of diagnosis. Therefore, we recommend that a structured approach based on previous studies and evidence should be used rather than one testing modality if there is high clinical suspicion of SARS-CoV-2 infection.
